# Fieberhafter papulovesikulärer Ausschlag bei einer Patientin mit Pyoderma gangraenosum

**DOI:** 10.1111/ddg.15887_g

**Published:** 2026-02-05

**Authors:** Anika Rajput Khokhar, Charlotte S. Wilm, Katharina Meier, Kamran Ghoreschi, Ulrike Blume‐Peytavi, Farzan Solimani

**Affiliations:** ^1^ Klinik für Dermatologie Venerologie und Allergologie Charité – Universitätsmedizin Berlin Körperschaft des öffentlichen Rechts Mitglied der Freien Universität Berlin und der Humboldt‐Universität zu Berlin; ^2^ Fachklinik Hornheide, Münster

Sehr geehrte Herausgeber,

Das Pyoderma gangraenosum (PG) ist eine seltene autoinflammatorische neutrophile Dermatose, die durch schnell wachsende, sehr schmerzhafte Ulzerationen meist an den unteren Extremitäten gekennzeichnet ist. Die Behandlung des PG zielt auf die Reduktion der Krankheitsaktivität und die Wundheilung ab. Therapiekonzepte basieren auf verschiedenen immunsuppressiven Medikamenten, darunter systemische Kortikosteroide, Ciclosporin und Tumornekrosefaktor (TNF)‐α‐Inhibitoren.^1^, ^2^ Die erforderliche dauerhafte immunsuppressive Behandlung und die großflächigen Wunden stellen für diese Patientengruppe ein Risiko für die Entwicklung von Infektionskrankheiten dar.^3^ Immunsupprimierte Patienten bedürfen einer besonderen Unterweisung und Sorgfalt in Bezug auf Impfungen und Reaktionen auf Infektionen, da *(1)* Infektionen aufgrund des immunsupprimierten Status einen schwereren Verlauf nehmen können und *(2)* Infektionen die zugrundeliegende entzündliche oder autoimmune Erkrankung verschlimmern können. Daher stellen Sicherheitsmaßnahmen, einschließlich präventiver Vorkehrungen wie Impfungen, einen entscheidenden Bestandteil der Behandlung dieser Patientengruppe dar.

Ein aktueller Fall aus unserer Klinik unterstreicht dieses Konzept nachdrücklich. Eine 21‐jährige Patientin mit einer Vorgeschichte eines Pyoderma gangraenosum stellte sich in unserer Abteilung mit Fieber und einem neuen polymorphen Exanthem vor. Sie zeigte kleine erythematöse, durchscheinende Vesikel und nekrotische Papeln, insbesondere im Gesicht, am Oberkörper und im Genitalbereich, begleitet von oralen Aphthen (Abbildung 1). Die Symptome hatten sich in den letzten drei Tagen verstärkt, verbunden mit starken Schmerzen im unteren Rücken. Die Patientin stammte ursprünglich aus der Ukraine, wo das Pyoderma gangraenosum erstmals diagnostiziert worden war. Sie erhielt eine immunsuppressive Therapie mit Ciclosporin (300 mg/Tag) in Kombination mit Prednison (10 mg/Tag). Anamnestisch war sie in der Vergangenheit nicht an Windpocken erkrankt. Sie konnte sich nicht an Impfungen in der Kindheit erinnern und besaß keinen Impfpass. Die Patientin wurde zur weiteren Diagnostik und Therapie stationär aufgenommen.

**ABBILDUNG 1:**
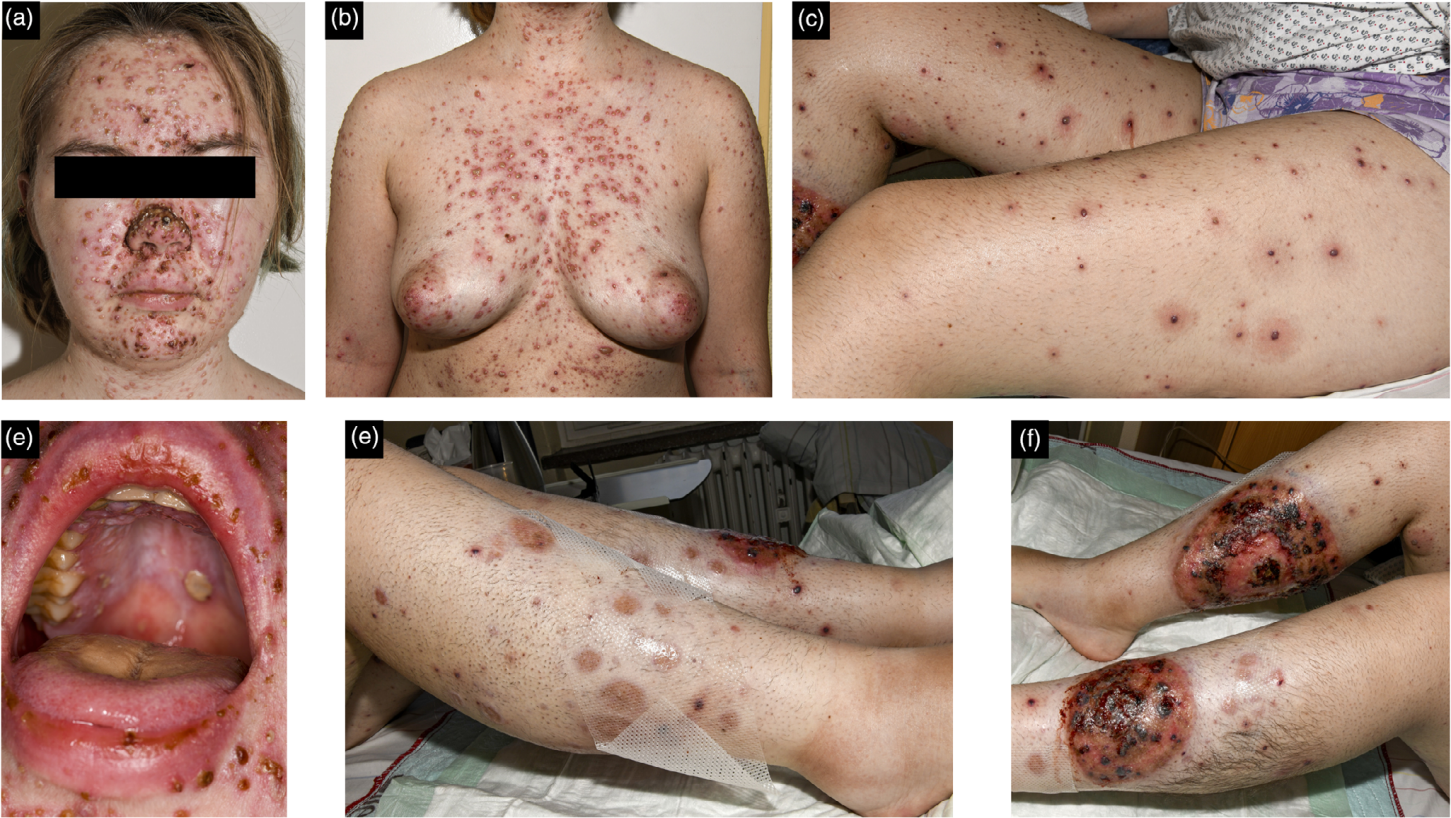
(a–d) Klinische Darstellung einer Infektion mit dem Varizella‐Zoster‐Virus. (e–f) Parainfektiöse Verschlimmerung eines Pyoderma gangraenosum.

Bei Verdacht auf eine Primärinfektion mit dem Varizella‐Zoster‐Virus (VZV) unter iatrogener Immunsuppression wurde eine antivirale Therapie mit Aciclovir (10 mg/kg Körpergewicht [KG]) eingeleitet und Ciclosporin pausiert. Die VZV‐Infektion wurde durch Polymerase‐Kettenreaktion (PCR) eines Hautabstrichs und serologischen Nachweis von IgM‐ und IgG‐Antikörpern bestätigt. Masern‐Antikörper waren nicht nachweisbar.

Aufgrund erhöhter Entzündungswerte – C‐reaktives Protein 259,3 mg/l (Referenzbereich < 5 mg/l) und Leukozyten 13,88/nl (Referenzbereich 3,90–10,50/nl) – leiteten wir zusätzlich eine breit angelegte antibiotische Therapie mit Piperacillin/Tazobactam (4,5 g alle 8 Stunden) ein. Infektionserkrankungen wie Hepatitis B oder C, Tuberkulose und HIV wurden ausgeschlossen. Da bei Erwachsenen mit einer VZV‐Infektion eine Lungenbeteiligung auftreten kann, führten wir eine Röntgen‐Thorax‐Untersuchung durch, die eine VZV‐Pneumonie ausschloss. Eine Urinanalyse und Blutkulturen gab es keinen Anhalt für weitere Infektionen. Während der VZV‐Infektion und des medikationsfreien Intervalls verschlechterte sich ihr PG rasch hinsichtlich Bezug auf Schmerzen, Größe und Anzahl der ulzerativen Läsionen. Aufgrund der gleichzeitigen VZV‐Infektion vermieden wir immunsuppressive Maßnahmen und begannen wegen der Exazerbation des Pyoderma gangraenosum eine intravenöse Immunglobulintherapie (2 g/kg KG über 5 Tage).^4^

Nach Abschluss der antiviralen Therapie und klinischer Kontrolle der VZV‐Infektion stellten wir die immunsuppressive Therapie auf orales Prednison (50 mg/Tag) und Infliximab (5 mg/kg Körpergewicht) um. Darüber hinaus traten neue Gelenkschmerzen und ‐schwellungen auf, mit nachweisbaren Antikörpern gegen citrullinierte Proteine (ACPA, 26,2 U/ml, Referenzbereich < 20 U/ml), jedoch ohne Erhöhung der Rheumafaktoren oder HLA‐B27‐Positivität. In Rücksprache mit unseren Rheumatologen vermuteten wir eine reaktive Arthritis und begannen eine Therapie mit Methotrexat (10 mg s.c./Woche), um Prednison zu reduzieren und die langfristige Einnahme hoher Steroiddosen zu vermeiden. Eine ganzheitliche Betreuung umfasste zusätzlich Schmerztherapie und psychosomatische Unterstützung.

Dieser Fall einer Varizelleninfektion bei einer jungen Patientin mit PG unterstreicht die Bedeutung eines aktuellen Impfstatus vor der Einleitung einer immunsuppressiven Therapie. Seit dem Jahr 2004 empfiehlt die *Ständige Impfkommission* (STIKO) die Varizellenimpfung für alle Kinder bis zum 18. Lebensjahr und für Erwachsene in besonderen Indikationen, einschließlich seronegativer Personen vor einer geplanten immunsuppressiven Therapie oder Organtransplantation. Die WHO empfiehlt diese Impfung nur in Ländern, in denen die Ressourcen ausreichen, um eine Durchimpfungsrate von mehr als 80 % zu erreichen und aufrechtzuerhalten, da ein niedrigerer Prozentsatz zu einer Verlagerung der Infektionen ins höhere Alter und damit zu einer höheren Morbidität und Mortalität führen kann. In der Ukraine wird die VZV‐Impfung nicht und auch in Polen wird sie nur für bestimmte Risikogruppen empfohlen. Im Gegensatz dazu wird die Varizellenimpfung beispielsweise in Frankreich, Griechenland und Luxemburg allgemein empfohlen und ist in Ungarn und Italien sogar verpflichtend.^5^ Außerdem sollte bei Immunsuppression und VZV‐Seropositivität oder nach einer Varizellenimpfung eine Gürtelroseimpfung in Betracht gezogen werden. Bei letzterer handelt es sich um einen rekombinanten Nicht‐Lebendimpfstoff, der während einer immunsuppressiven Therapie verabreicht werden kann, während es sich bei dem VZV‐Impfstoff um ein abgeschwächtes Lebendvirus handelt und die Impfung mit einem Sicherheitsabstand vor Therapiebeginn erfolgen muss.

In unserem Fall führten Faktoren wie der Verlust des Impfausweises, Sprachbarriere und ein erschwerter Zugang zu medizinischer Versorgung einschließlich eines Hausarztes zu einem suboptimalen Impfstatus während der immunsuppressiven Behandlung und einer schweren Varizelleninfektion. Verstärkte Anstrengungen, wie international standardisierte Impfpläne für Kinder, sind erforderlich, um immunsupprimierte und transplantierte Hochrisikogruppen zu schützen.

## DANKSAGUNG

Open access Veröffentlichung ermöglicht und organisiert durch Projekt DEAL.

## INTERESSENKONFLIKT

Keiner.
